# Polysaccharides From *Chrysanthemum morifolium* Ramat Ameliorate Colitis Rats via Regulation of the Metabolic Profiling and NF-κ B/TLR4 and IL-6/JAK2/STAT3 Signaling Pathways

**DOI:** 10.3389/fphar.2018.00746

**Published:** 2018-07-10

**Authors:** Jin-Hua Tao, Jin-Ao Duan, Wei Zhang, Shu Jiang, Jian-Ming Guo, Dan-Dan Wei

**Affiliations:** ^1^School of Pharmacy, Nantong University, Nantong, China; ^2^Jiangsu Collaborative Innovation Center of Chinese Medicinal Resources Industrialization, Nanjing University of Chinese Medicine, Nanjing, China

**Keywords:** Chrysanthemum polysaccharides, colitis, metabonomics, signaling pathway, IBD

## Abstract

Studies have indicated that Chrysanthemum polysaccharides (CP) could prominently ameliorate colitis rats, but its possible mechanism remains unclear. In this study, the underlying mechanism of CP was explored by the metabolic profiling analysis and correlated signaling pathways. TNBS/ethanol induced colitis was used to investigate the intervention efficacy following oral administration of CP. The levels of cytokines such as TNF-α, IL-6, IFN-γ and IL-1β, and the activities of SOD, MPO, and MDA were determined. We also performed western-blot for p65, TLR4, p-JAK2, and STAT3 protein expression in the colon tissue to probe their mechanisms of correlated signaling pathways. What’s more, the metabolic changes in plasma and urine from colitis rats were investigated based on UPLC-Q-TOF/MS combined with Metabolynx^TM^ software. The potential biomarkers and metabolic pathways were also tentatively confirmed. The metabolic profiles of plasma and urine were clearly improved in model rats after oral administration of CP. Thirty-two (17 in serum and 15 in urine) potential biomarkers were identified. The endogenous metabolites were mainly involved in linoleic acid, retinol, arachidonic acid, glycerophospholipid and primary bile acid metabolism in plasma, and nicotinate and nicotinamide, ascorbate and aldarate, histidine and β-alanine metabolism in urine. After polysaccharides intervention, these markers turned back to normal level at some extent. Meanwhile, the elevated expression levels of pp65, TLR4, p-STAT3, and p-JAK2 were significantly decreased after treatment. Results suggested that CP would be a potential prebiotics for alleviation of TNBS-induced colitis. The study paved the way for the further exploration of the pathogenesis, early diagnosis and curative drug development of the colitis.

## Introduction

Inflammatory bowel disease, which is characterized by chronic and progressive inflammation in the gastrointestinal tract, is a chronic recurring condition with a high incidence of morbidity, classified into two distinct clinical forms, ulcerative colitis and Crohn’s disease. IBD manifests some symptoms which includes weight loss, severe diarrhea, debilitating abdominal pain and bloody stools, leading to impairment in quality of life. Patients with IBD also have a significantly increased risk of having a related disease such as colon cancer (Pizzi et al., 2006; Walker et al., 2011). So far, the pathogeny of IBD remains unknown, although several factors have been related to the development of disease, including genetics, abnormal immune regulation, barrier dysfunction, environmental exposures and the changes of the gut microbial community (Packey and Sartor, 2008; Mar et al., 2014; Tao et al., 2017). Dysregulation of immune response of intestinal bacterial flora was associated with the development of IBD (Junghee et al., 2009). The innate immune system could recognize the presence of bacterial invasion through PRRs in the intestinal epithelial cells, and the major PRRs are TLRs (Fort et al., 2005). Out of all the TLRs, induction of TLR4 isoform is implicated in IBD pathogenesis (Venkatesh et al., 2014). Convincing researches have indicated that LPS, a major component of Gram-negative bacterial cytoderm, binds to TLR4 and triggers MyD88-mediated signaling cascades activation of NF-κB, and eventually leads to inflammatory response (Siddique and Khan, 2011; Jang et al., 2013). TLR4 is generally expressed at low level in the intestinal mucosa but is notably up-regulated in IBD patients and colitis rats which implied its important role in the pathogenesis of IBD (Sun et al., 2016). IBD is usually accompanied by a typical imbalance of pro-inflammatory and anti-inflammatory signaling pathways in the intestine. NF-κB is a major regulator of gene transcription in these pathways. The disorder of the NF-κB signaling pathway usually causes overzealous inflammation in patients with IBD (Tsang et al., 2015; Wang et al., 2017). Various inflammatory cytokine genes that involved in the etiologies of IBD, including IL-1β, IL-2, TNF-α and IL-6, have NF-κB binding sites and are transcriptionally regulated by NF-κB (Baldwin, 1996; Barnes and Karin, 1997).

The type of metabolites and changes in metabolic profiles could serve as good indicator of cytokines-mediated inflammatory processes in IBD. Metabolomics is a systems biology approach (Holmes et al., 2008; Su et al., 2015) that measures the dynamic multi-parametric metabolic responses of living systems to pathophysiologic stimuli or genetic modification (Nicholson et al., 2008) by the simultaneous and non-targeted analysis of dynamic changes of endogenous metabolites in complex biological matrices (Phua et al., 2013). Changes of metabolites with low molecular weight usually reflect the end-results of the genomic and protein perturbations in disease and are closely related to phenotypic changes. Additionally, through the identification of biomarkers, analysis of metabolic pathways, discovery of drug-target interactions, etc., it is also possible to elucidate the pathogenesis of diseases and the action mechanisms of therapy. Therefore, metabonomics has attracted an interest for investigating the IBD and evaluating drug treatment outcomes (Holmes et al., 2008; Schicho et al., 2010; Lin et al., 2011; Su et al., 2015).

Unfortunately, therapeutic options currently available for the treatment of IBD are largely limited to symptoms of the disease, with some exceptions to the molecular pathways associated with intestinal inflammation (Jobin, 2010; Okafor et al., 2013). Despite advances in therapeutics, some patients didn’t respond to treatment or suffered from side effects or complications (Dou et al., 2014). And so, recent researches have focused on the use of natural products or dietary supplements as alternatives to patients who are unresponsive or unwilling to take commonly medications (Recio et al., 2012). As a medicinal and edible homologous plant, chrysanthemum was reported to have strong heat-clearing and detoxifying ability, which can effectively treat infectious diseases and some inflammatory diseases such as influenza, colitis, stomatitis, and can also be used clinically to treat cancer (Kim et al., 2013; Luyen et al., 2015; Zheng et al., 2015; Tao et al., 2016, 2017). Our previous studies showed that CP have a significant protective effect on colitis mice by increasing SCFAs production (Tao et al., 2016). However, the mechanism of colonic fermentation is not yet clear, and the effect of CP on the intestinal microbiota is also limited (Tao et al., 2017).

In this study, TNBS/ethanol induced colitis was used to investigate the intervention efficacy after oral administration of CP. The levels of cytokines such as TNF-α, IFN-γ, IL-6 and IL-1β, and the activities of SOD, MDA, and MPO were determined. To elucidate the mechanism, the metabolic changes in plasma and urine from TNBS-induced colitis rats were investigated based on UPLC-Q-TOF/MS combined with Metabolynx^TM^ software. The potential biomarkers and metabolic pathway were also tentatively identified. The data on the basis of this work would be useful to clarify the potential mechanism of CP to improve and alleviate colitis.

## Materials and Methods

### Animals

Male SD rats (220 ± 20 g) from the Vital River Laboratory Animal Technology Co., Ltd. (Beijing, China) were accommodated in the specific pathogen-free animal center with an air-conditioned animal quarter with 12 h light/12 h dark cycle and a constant temperature of 22 ± 2°C. The rats were allowed to acclimate for 7 days prior to experiments and were fed with standard food and water *ad libitum*.

### Chemical Regents

2, 4, 6-trinitrobenzene sulfonic acid and sulfasalazine were bought from MP Biomedical (Aurora, OH, United States) and Yifeng Pharmacy (Nanjing, China), respectively. ELISA kits (IL-6, IL-1β, IFN-γ, and TNF-α) were purchased from Nanjing Jiancheng Bioengineering Institute Co., Ltd. CP were prepared according to our previous methods (Schicho et al., 2010; Tao et al., 2017).

### Induction of Colitis Rat Model and Treatment Protocol

The rats were anesthetized with chloral hydrate and then administered with TNBS/ethanol solution into the colon from the rectum. 24 h later, all the rats were randomly assigned to six groups. N, receiving normal saline; M, receiving ethanol vehicle with TNBS; PP, receiving SASP 0.5 g/kg; HP, MP and LP, receiving CP 200, 100, and 50 mg/kg, respectively (Scheiffele and Fuss, 2002; Tao et al., 2017) (the details were listed in **Supplementary Text S1**).

### Sample Collection and Preparation

After the last treatment day, all experimental rats were put in the metabolism cages for urine collection, and blood samples were gathered into the tubes containing EDTA-2Na on the 16th day from carotid artery after ether deep anesthesia. After collection of urine and blood, all rats were sacrificed. The colon was dissected and incised longitudinally along the mesenterium, rinsed with saline, and then used to assess colon mucosal damage and biochemical tests.

The urine and blood samples were thawed under room temperature prior to analysis. 200 μL of plasma and urine were, respectively, added to 600 μL of acetonitrile, vortexed for 30 s and centrifuged at 13,000 g at 4°C for 10 min to obtain a supernatant. The plasma and urine supernatants were evaporated to dryness in a 37°C water bath under a gentle stream of nitrogen. The residues were reconstituted in 200 μL of a 70% acetonitrile-water solution mobile phase, filtered through a 0.22 μm membrane filter for UPLC/MS analysis (Su et al., 2015).

### UPLC-Q-TOF/MS Analysis Conditions

Metabolite separation was performed using a Waters Acquity^TM^ UPLC system equipped with a Waters Xevo^TM^ G2 Q/TOF-MS. 2 μL sample solution was injected into an ACQUITYTM UPLC BEH C18 (100 mm × 2.1 mm, 1.7 μm) with the flow rate was 0.4 mL/min at 35°C. The optimal mobile phase consisted of water (A) 0.1% formic acid and (B) acetonitrile. The optimized UPLC elution conditions for serum were: 0 ∼ 3 min, 95% ∼ 55% A; 4 ∼ 13 min, 55% ∼ 5% A; 13 ∼ 14 min, 5% A; The optimized UPLC elution conditions for urine were: 0 ∼ 8 min, 95% ∼ 70% A; 8 ∼ 11 min, 70% ∼ 30% A; 11 ∼ 13 min, 30% ∼ 5% A; 13 ∼ 14 min, 5% A.

Mass spectrometry detection was performed using a quadrupole and orthogonal acceleration time-of-flight tandem mass spectrometer. Data were collected in centroid mode from 100 to 1000 m/z. For both positive and negative electrospray modes, the capillary and cone voltage were set at 3.0 kV and 30 V, respectively. The desolvation gas was set to 600 L/h at a temperature of 350°C, the cone gas was set to 50 L/h and the source temperature was set to 120°C (Zhao et al., 2012).

### Determination of Cytokines (TNF-α, IFN-γ, IL-6, and IL-1β)

50 mg of colon tissue was extracted with 500 μL of guanidine hydrochloride and Tris-HCl (50 mM, pH 8.0) containing protease inhibitors. The extracts were then centrifuged at 13,000 *g* for 10 min at 4°C. The cytokines were then analyzed by ELISA kits using the supernatant fraction according to the manufacturer’s instructions.

### MPO, SOD, and MDA Assay

The MPO, SOD, and MDA activity was determined according to the respective assay kit. The supernatant of colon tissue were obtained by the method described above.

Myeloperoxidase which is often over expressed in numerous inflammatory diseases, including IBD (Lau and Baldus, 2006; Pattison and Davies, 2006), has the potential to serve as a viable, non-invasive fecal biomarker for assessing IBD status. MDA is a product of lipid peroxidation and degradation, which reacts with TBA to form a red product that absorbs at a wavelength of 532 nm (Su et al., 2015).

### Metabolomic Data Processing and Multivariate Data Analysis

All of the UPLC/MS data acquisition and analyses were controlled by Waters Mass Lynx v4.1 and MarkerLynx software. The multivariate data matrix was analyzed using *E*Zinfo software. Unsupervised segregation was examined with a PCA using Pareto-scaled data. A PLS-DA and an OPLS-DA were used to identify the various meta∖bolites responsible for the separation between the normal and model groups (Su et al., 2013). Potential biomarkers were extracted from the S-plots that were constructed following the OPLS-DA analysis, and the biomarkers were chosen based on their contribution to the variation and correlation within the dataset. The VIP in the projection value was a weighted sum of squares of the PLS weights, and the variables with VIP > 1 were considered to be influential for the separation of samples in the score plots generated from PLS-DA analysis (Salliot and Van, 2009; Su et al., 2015).

### Biomarker Identification and Metabolic Pathway Analysis

Potential biomarkers were corroborated by comparing their chromatographic retention times, mass spectra with the standards and a full spectral library with MS/MS data acquired in the negative and/or positive ion modes. MS/MS fragment ions were analyzed by the mass fragment application manager MassLynx v4.1with chemically intelligent peak-matching algorithms. Then, based on the above information, multiple databases were searched and potential metabolic pathways were analyzed by METLIN^1^, Human Metabolome Database^2^, and MetPA^3^ based on the KEGG^4^.

### Quantitative Real-Time Polymerase Chain Reaction (qPCR)

Total RNA was isolated from colon tissues using Trizol reagents following the manufacturer’s specifications [*TranScript*^®^ All-in-One First-Strand cDNA Synthesis SuperMix for qPCR (One-Step gDNA Removal)]. Real-time quantitative PCR was performed using SYBR Green Master mix and Rox reference dye. Transcript levels were quantified by the ΔΔ*C*t value method (Pan et al., 2000; Su et al., 2015). Calculation was done using the *C*t value of GAPDH to normalize the *C*t value of target genes in each sample to obtain the ΔΔ*C*t value, which was then used to compare among different samples (Huang et al., 2016). PCR products were analyzed by gel electrophoresis on 1.5% agarose gel and the specificity of amplification was confirmed by the melting curves. The primer sequences of target genes were listed as **Supplementary Table S1**.

### Western Blot Analysis for NF-κB p65, pp65, TLR4, STAT3, p-STAT3, and JAK2

Colonic tissues were disrupted by homogenization in lysis buffer containing protease inhibitor and the supernatants (total protein) were collected after centrifugation. The protein lysates were separated by 10% SDS-PAGE, transferred to PVDF membranes, and blocked with 0.05% (w/v) Tween 20 and 5% (w/v) BSA over night at 4°C. The immunoblots were incubated with primary antibodies at 1:1000 dilution with 1% BSA for 3 h at 4°C. Subsequently, the immunoblots were incubated with the secondary antibody conjugated with IgG-HRP at room temperature for 1 h and then washed with TBST in triplicate, and then the bands were detected using chromogenic substrate. β-actin was used as loading control.

### Statistical Analysis

The experimental data were analyzed by GraphPad PrismV5 and the results were expressed as mean ± standard deviation. Comparison of the mean number of groups using one-way ANOVA and Tukey’s true significance difference (HSD) *post hoc* test (Wu et al., 2016; Tao et al., 2017).

## Results

### Effection of Chrysanthemum Polysaccharides on TNBS-Induced Colitis

This study established a TNBS-induced colitis model and was successfully used to evaluate the amelioration of CP (Tao et al., 2017). Physiological and pathological changes of colitis rats after oral administration of CP have been detailly described in our previous reported protocol (**Supplementary Text S1** and **Supplementary Figure S1**). The above results indicated that CP strongly protected and notably alleviated the TNBS-induced colitis (Tao et al., 2016).

### Effects of CP on MPO, SOD, and MDA Activities in Colon Tissues

**FIGURE 1:**
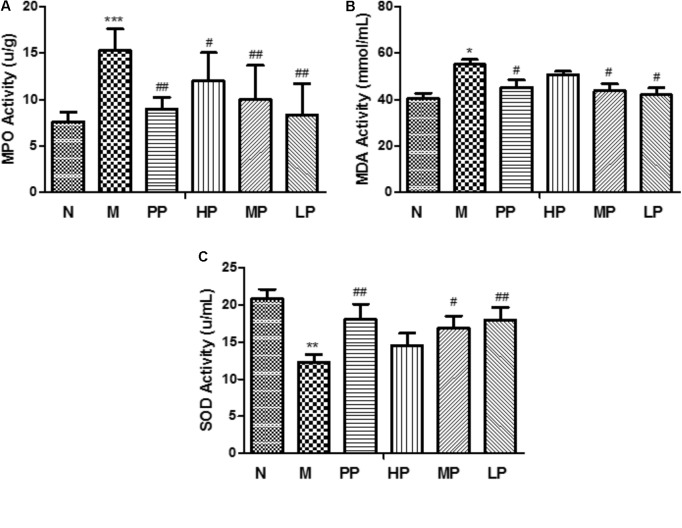
MPO **(A)**, SOD **(B)**, and MDA **(C)** activities in colonic tissue of different rats. N vs. M, ^∗^*p* < 0.05, ^∗∗^*p* < 0.01, and ^∗∗∗^*p* < 0.001. PP, HP, MP, LP vs. M, ^#^*p* < 0.05 and ^##^*p* < 0.01.

Myeloperoxidase is an enzyme which found primarily in neutrophils and has been used as a quantitative indicator of inflammation and neutrophil infiltration in the colonic mucosa. The decrease of MPO activity could be interpreted as the performance of the anti-inflammatory properties (Xing et al., 2012; Chen et al., 2015). In this study, as shown in **Figure 1A**, compared to the normal group, the activity of MPO was dramatically increased (15.31 ± 2.32 U/g vs. 7.62 ± 1.03 U/g) in TNBS-induced colitis rats (*p* < 0.001). However, CP with different doses (high dosage, HP 200 mg/kg, middle dosage, MP 100 mg/kg, low dosage, LP 50 mg/kg) and SASP (0.5 g/kg) could significantly inhibit the activity of MPO in the model rats.

Malondialdehyde is a peroxidation product by the attack of free radicals to lipids and its level represents the intensity of body injury (Su et al., 2015). The levels of MDA in colonic tissue had the same trends with the activity of MPO in colitis rats than that of normal rats, and interventional effects of CP were seen in **Figure 1B**.

Superoxide dismutase is an important antioxidant enzyme in organism, which can effectively scavenge oxygen free radicals and inhibit lipid in colon tissue qualitative peroxidation, and can stabilize the cell membrane and produce specific responses to certain inflammatory stimuli. The level of SOD is the main indicator to reflect the function of cell membrane and the body’s anti-inflammatory reaction (Halliwell, 1996). The SOD activity in colonic tissue indicates the intensity of the antioxidant defense system in UC. Tissue SOD may protect against oxidative stress caused by ROS. Because the antioxidant defense system in chronically inflamed tissues is impaired, this action might confer susceptibility to DNA damage and mutation, resulting in the development of carcinoma (Korenaga et al., 2002). In this study, the SOD activity was obviously decreased after TNBS induction compared with normal group (12.33 ± 1.01 vs. 20.86 ± 1.24 U/mL). After oral administration of with middle and low doses of CP, SOD activity was recovered notably (*p* < 0.05) (**Figure 1C**).

### Effects of CP on the Levels of TNF-α, IFN-γ, IL-6, and IL-1β

**FIGURE 2:**
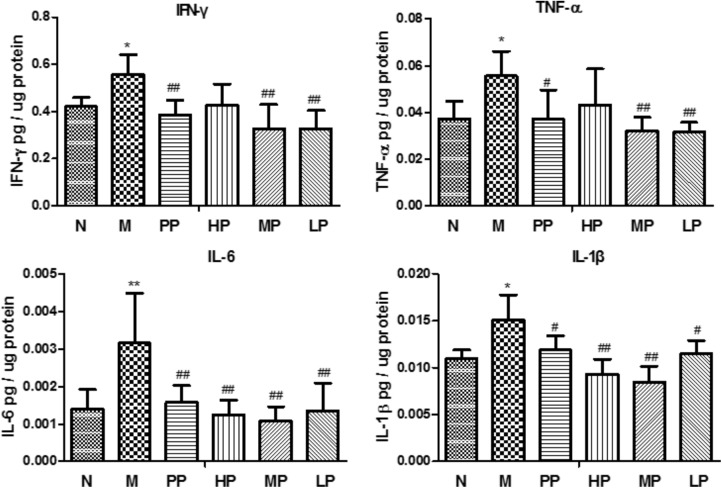
Chrysanthemum polysaccharides (CP) regulated pro-inflammatory cytokines in colons mucosa of different groups. Cytokine levels in colonic proteins were assessed by ELISA. All data were presented as mean ± SD. *N* = 6 for each group. N vs. M, ^∗^*p* < 0.05, ^∗∗^*p* < 0.01, and ^∗∗∗^*p* < 0.001. PP, HP, MP, LP vs. M, ^#^*p* < 0.05, ^##^*p* < 0.01, and ^###^*p* < 0.001.

As depicted in **Figure 2**, TNBS/ethanol instillation significantly promoted the levels of TNF-α, IFN-γ, IL-6, and IL-1β in rats. In contrast with the colitis group, different doses of CP remarkably attenuated the levels of these pro-inflammatory cytokines in various degrees (*p* < 0.05), which indicated that CP effectively reduced the pro-inflammatory factors in colitis rats.

### Chrysanthemum Polysaccharides Inhibited mRNA Levels of TLR4, NF-κB, IL-6, STAT3, and JAK2 in TNBS-Induced Colitis Rats

**FIGURE 3:**
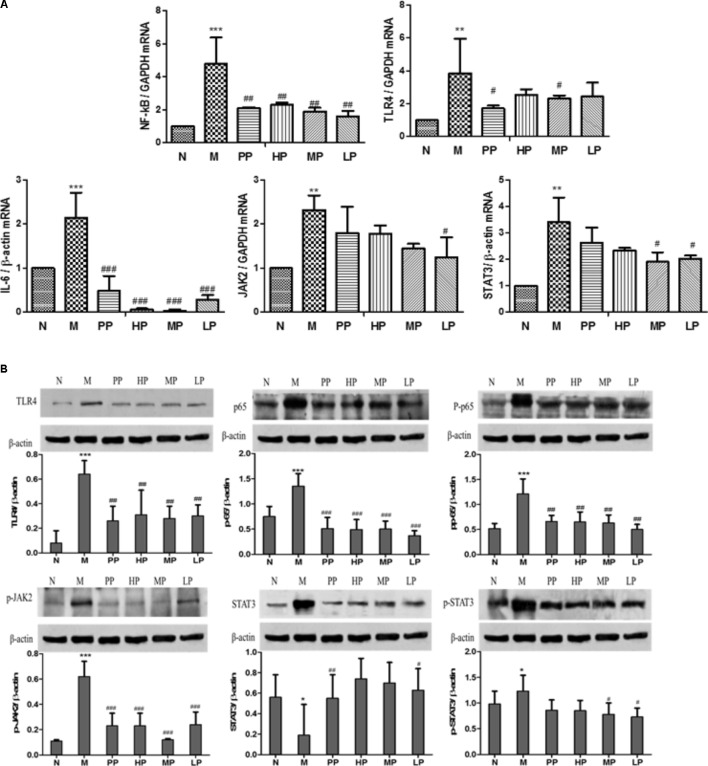
**(A)** Effects of CP on mRNA levels of TLR4, NF-κB, IL-6, STAT3, and JAK2 in TNBS-induced colitis. **(B)** Effects of CP on the expression of p65, pp65, TLR4, STAT3, p-STAT3, and JAK2 in colitis rats. N vs. M, ^∗^*p* < 0.05, ^∗∗^*p* < 0.01, ^∗∗∗^*p* < 0.001. PP, HP, MP, LP vs. M, ^#^*p* < 0.05, ^##^*p* < 0.01, ^###^*p* < 0.001.

As shown in **Figure 3A**, compared with the normal group, mRNA levels of TLR4, NF-κB, IL-6, STAT3, and JAK2 in colitis rats remarkably increased (*P* < 0.001, *P* < 0.01). However, when treated with different dosages of CP (HP, 200 mg/kg, MP, 100 mg/kg, LP, 50 mg/kg) and Sulfasalazine (0.5g/kg), mRNA expressions of these factors decreased in varied degrees (Tao et al., 2017). The relative mRNA levels of NF-κB and IL-6 significantly decreased by each dosage of CP (*P* < 0.01, *P* < 0.001). The expressions of TLR4 and JAK2 mRNA were inhibited by MP and LP, respectively. Both MP and LP could reduce STAT3 mRNA notably (*P* < 0.05).

### Chrysanthemum Polysaccharides Suppressed the Expression of p65, pp65, TLR4, STAT3, p-STAT3, and JAK2 in TNBS-Induced Colitis Rats

An attenuated trend of the expression of p65, pp65, TLR4, STAT3, p-STAT3, JAK2 in TNBS-induced colitis rats was observed in the groups treated with CP. Colitis rats exhibited a remarkable tendency to up-regulate the above target proteins (*P* < 0.05, *P* < 0.01), while CP could notably lower the levels of pp65, TLR4, p-STAT3, and p-JAK2 (*P* < 0.05, *P* < 0.01, *P* < 0.001). As shown in **Figure 3B**, the relative pp65, TLR4, and p-JAK2 levels in TNBS-induced colitis rats were diminished significantly after treatment of HP, MP, and LP. However, there was no significant change in the expression of STAT3 and p-65. The results above illustrated that CP could retrace the expression of target proteins in NF-κB/TLR4 and IL-6/JAK2/STAT3 signaling pathways.

### LC-MS Analysis of Metabolic Profiling

**FIGURE 4:**
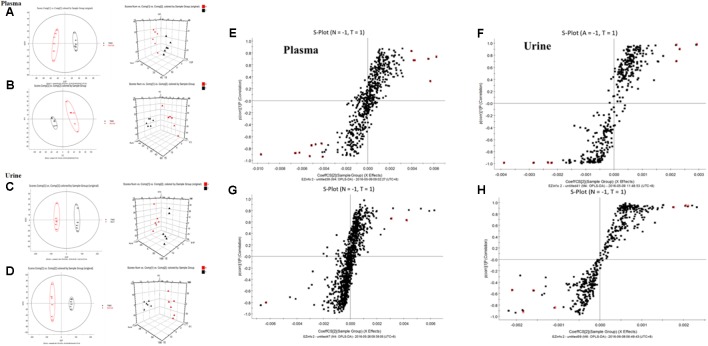
Principal component analysis (PCA) results between colitis and normal rats in positive and negative modes. **(A)** 2-D plot of plasma and 3D PLS-DA scores plot of LC-MS spectral data in ESI^-^. **(B)** 2-D plot of plasma and 3D PLS-DA scores plot of LC-MS spectral data in ESI^+^. **(C)** 2-D plot of urine and 3D PLS-DA scores plot of LC-MS spectral data in ESI^-^. **(D)** 2-D plot of urine and 3D PLS-DA scores plot of LC-MS spectral data in ESI^+^; S-plot of OPLS-DA model for colitis vs. normal group; **(E)** plasma, ESI^-^; **(F)** Urine, ESI^-^; **(G)** plasma, ESI^+^; **(H)** Urine, ESI^+^.

All the data containing the retention time, peak intensity and exact mass were imported into the Masslynx^TM^ software for multiple statistical analyses. Typical based peak intensity chromatograms of serum and urine samples, derived from normal controls (N) and colitis rats (M) in positive and negative modes, were presented as **Supplementary Figure S2**. Based on the data from the two groups, plasma or urine sample were divided into blocks between model and normal rats using the unsupervised PCA model to determine the metabolic changes and characterize the metabolite profile (**Figures 4A–D**). The supervised OPLS-DA with 100% sensitivity and no less than 95% specificity showed a better discrimination between the two groups (**Figure 4**), which demonstrated that the colitis model was successfully established. And subsequently, the OPLS-DA score plot (**Figures 4E–H**) was used to screen potential metabolites related to colitis progress (Cai et al., 2018).

### Identification of Potential Biomarkers and Intervention of Chrysanthemum Polysaccharides

The UPLC-Q-TOF/MS analysis provided the retention time, precise molecular mass and the fragments of corresponding production for the structural identification of metabolites. According to the precise molecular mass, the potential element composition was predicted and potential molecular formula could be searched out based on the KEGG^5^ and Human Metabolome Database^6^ (Cai et al., 2018). Thirty-two potential biomarkers (17 in serum and 15 in urine) were ultimately identified by comparing with authentic standards or based on the protocol detailed above method. The information about the detected endogenous metabolites was summarized in **Table 1**.

**Table 1:** The identified and change trend of the potential biomarkers between colitis and normal rats.

Number	tR/min	Actual-mass	Formula	Ion mode	Metabolite	Trend	VIP	KEGG	HMDB	Source
1	3.21	246.0921	C5H12O7P2	-	Dimethylallylpyrophosphate	↓	2.25	C00235	HMDB01120	Urine
2	4.21	108.0948	C6H4O2	-	1,2-Benzoquinone	↑	1.95	C02351	HMDB12133	Urine
3	3.82	114.1026	C4H6N2O2	-	Dihydrouracil	↓	1.02	C00429	HMDB00076	Urine
4	4.45	176.1241	C6H8O6	-	D-Glucurono-6,3-lactone	↑	1.53	C02670	HMDB06355	Urine
5	6.04	122.1246	C6H6N2O	-	Niacinamide	↓	1.25	C00153	HMDB01406	Urine
6	3.71	257.2213	C8H20NO6P	-	Glycerophosphocholine	↓	1.34	C00670	HMDB00086	Urine
7	3.17	161.1558	C6H11NO4	-	Aminoadipic acid	↓	2.03	C00956	HMDB00510	Urine
8	3.15	194.1394	C6H10O7	-	D-Glucuronic acid	↓	2.04	C00191	HMDB00127	Urine
9	14.7	140.1399	C6H8N2O2	+	Methylimidazoleacetic acid	↑	1.93	C05828	HMDB02820	Urine
10	12.81	138.124	C6H6N2O2	+	Urocanic acid	↓	1.64	C00785	HMDB00301	Urine
11	14.65	148.18	C5H8O3S	+	2-Oxo-4-methylthiobutanoic acid	↑	3.35	C01180	HMDB01553	Urine
12	3.17	337.2198	C11H16NO9P	+	Nicotinic acid mononucleotide	↓	2.64	C01185	HMDB01132	Urine
13	3.7	339.1959	C9H14N3O9P	+	5-amino-1-(5-phospho-D-ribosyl)imidazole-4-carboxylate	↓	2.52	C04751	HMDB06273	Urine
14	12.81	184.1507	C5H15NO4P	+	Phosphorylcholine	↓	1.63	C00588	HMDB01565	Urine
15	4.54	129.157	C6H11NO2	+	L-Pipecolic acid	↓	1.70	C00408	HMDB00716	Urine
16	11.27	280.4455	C18H32O2	-	Linoleic acid	↓	7.73	C01595	HMDB00673	Plasma
17	10.73	284.4357	C20H28O	-	Retinal	↓	4.11	C00376	HMDB01358	Plasma
18	11.01	304.4669	C20H32O2	-	Arachidonic acid	↓	5.77	C00219	HMDB01043	Plasma
19	10.04	302.451	C20H30O2	-	Retinyl ester	↓	3.05	C02075	HMDB03598	Plasma
20	7.54	481.309	C26H45NO6S	-	Taurochenodesoxycholic acid	↓	3.43	C05465	HMDB00951	Plasma
21	14.71	182.08	C4H9NO2Se	-	Se-Methylselenocysteine	↑	1.24	C05689	HMDB04113	Plasma
22	3.68	408.5714	C24H40O5	-	Cholic acid	↓	1.96	C00695	HMDB00619	Plasma
23	3.9	465.6227	C26H43NO6	-	Glycocholic acid	↑	1.43	C01921	HMDB00138	Plasma
24	7.05	318.4504	C20H30O3	-	Leukotriene A4	↑	2.08	C00909	HMDB01337	Plasma
25	5.95	392.572	C24H40O4	-	Chenodeoxycholic acid	↓	1.98	C02528	HMDB00518	Plasma
26	14.45	466.717	C27H46O4S	-	Cholesterol sulfate	↑	1.10	C18043	HMDB00653	Plasma
27	10.31	523.6832	C26H54NO7P	-	LysoPC (18:0)	↓	1.07	C04230	HMDB10384	Plasma
28	4.58	449.6233	C26H43NO5	-	Chenodeoxycholic acid glycine conjugate	↓	1.02	C05466	HMDB00637	Plasma
29	9.75	165.1461	C8H7NO3	-	Formylanthranilic acid	↓	1.01	C05653	HMDB04089	Plasma
30	7.89	183.2044	C9H13NO3	+	Normetanephrine	↑	1.09	C05589	HMDB00819	Plasma
31	1.71	187.1947	C11H9NO2	+	Indoleacrylic acid	↓	1.73	C00331	HMDB00734	Plasma
32	8.29	329.5179	C19H39NO3	+	Dihydroceramide	↑	1.06	C12126	HMDB06752	Plasma

**FIGURE 5:**
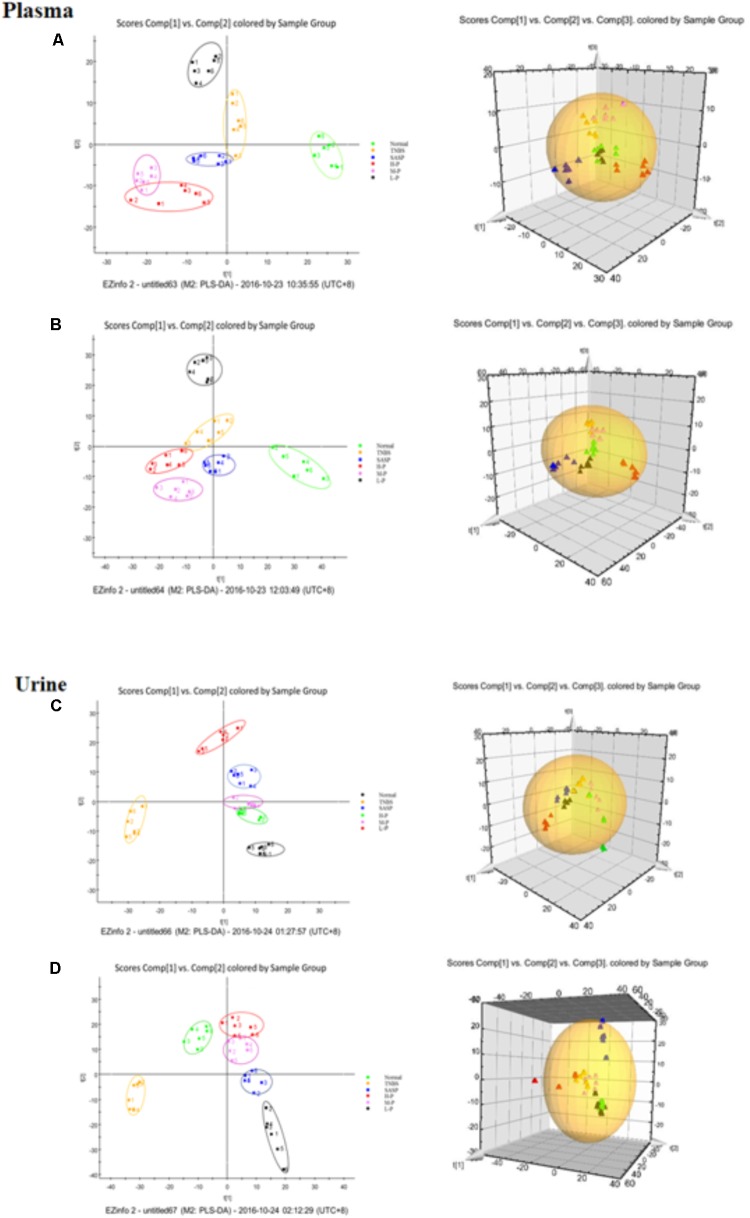
Partial least squares discriminant analysis (PLS-DA) plot in normal rats, colitis rats, SASP group and administration group rats. **(A)** plasma, ESI^-^; **(B)** plasma, ESI^+^; **(C)** urine, ESI^-^; **(D)** urine, ESI^+^.

**FIGURE 6:**
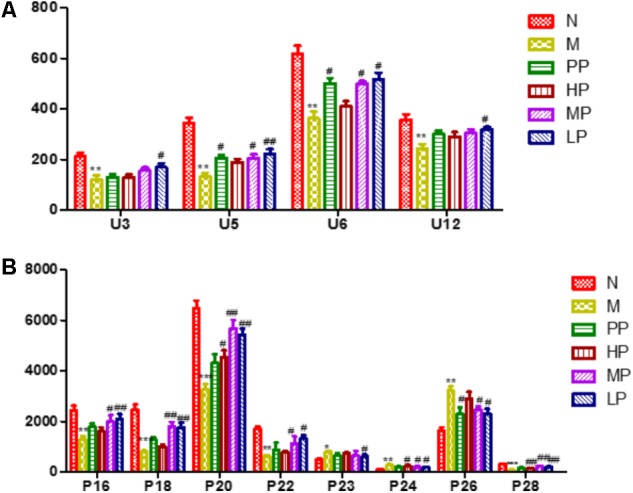
The relative quantities of target endogenous metabolites affected by CP. **(A)** Urine; **(B)** plasma. The corresponding markers represented to the **Table 1**. N vs. M, ^∗^*p* < 0.05, ^∗∗^*p* < 0.01, ^∗∗∗^*p* < 0.001. PP, HP, MP, LP vs. M, ^#^*p* < 0.05, ^##^*p* < 0.01, ^###^*p* < 0.001.

To investigate the amelioration and action mechanism of CP for curing colitis rats, PLS-DA model analysis was built to obtain the changes during the normal groups, TNBS-induced groups, SASP groups and administration groups. The variations of plasma and urine metabolic profiling of SASP groups and different dosages of CP were restored to the levels of normal controls in the varied degree (**Figure 5**). Furthermore, the relative quantities of eight potential biomarkers in plasma and four in urine were significantly affected by CP. Especially, the middle and low dosage groups were restored to a control-like level (**Figure 6**). These changes may not immediately in response to therapeutic effects of CP for the colitis rats, but they are produced by the perturbation of CP from the organism. The contents of the potential biomarkers in **Table 1** were considered as biomarkers for therapeutic efficacy.

**FIGURE 7:**
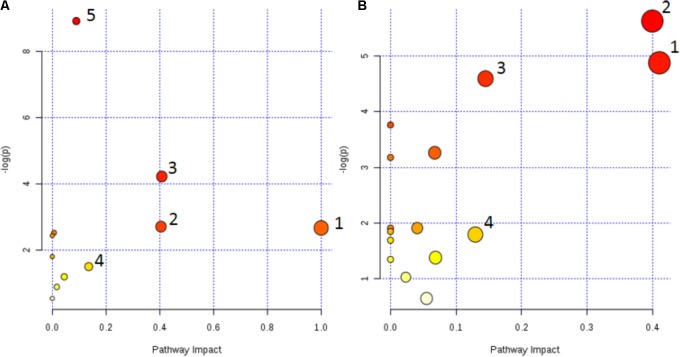
Metabolic pathways involved in potential markers in plasma and urine. **(A)** (1) Linoleic acid metabolism, (2) Retinol metabolism, (3) Arachidonic acid metabolism, (4) Glycerophospholipid metabolism, and (5) Primary bile acid biosynthesis. **(B)** (1) Nicotinate and nicotinamide metabolism, (2) Ascorbate and aldarate metabolism, (3) Histidine metabolism, and (4) β-Alanine metabolism.

The metabolic pathway was established by importing the potential metabolites into the web-based database MetPA. The pathway impact value calculated from pathway to topology analysis with MetPA above 0.1 was screened out as the potential target pathway (Cai et al., 2018). As shown in **Figure 7A**, among the eleven pathways in plasma, (1) Linoleic acid metabolism, (2) Retinol metabolism, (3) Arachidonic acid metabolism, (4) Glycerophospholipid metabolism and (5) Primary bile acid biosynthesis with the impact value 1.0, 0.41, 0.40, 0.14, and 0.10, respectively, were filtered out as the most important metabolic pathways. In **Figure 7B**, 15 pathways in urine mainly involving (1) Nicotinate and nicotinamide metabolism, (2) Ascorbate and aldarate metabolism, (3) Histidine metabolism and (4) β-Alanine metabolism with the impact value 0.41, 0.4, 0.15, and 0.13, respectively, were filtered out. Results indicated that these target pathways showed the marked perturbations over the time-course of the treatment and could contribute to development of colitis.

## Discussion

Inflammatory bowel disease is a chronic, relapsing disease of which the etiology remains essentially unclear. However, the results from many studies in humans and animal models indicate that it is related to an abnormal immune response in the gastrointestinal tract (Miao et al., 2015). At present, lots of researches have confirmed that the intestinal microflora dysbiosis is closely related to the gastrointestinal disease (Waidmann et al., 2003; Walker et al., 2011). Patients with gastrointestinal disease usually have the phenomenon of intestinal flora imbalance. Under the stimulation of various microbial antigens, the immune system *in vivo* is activated, resulting in imbalance of cytokine production and secretion, followed by activation of various inflammatory cells and further chronic inflammatory response of intestinal tissue (Danese and Fiocchi, 2006). Our previous studies have demonstrated that the intestinal flora structure of rats with colitis remarkably changed. The abundances of some Gram-negative pathogens such as *Escherichia*, *Enterococcus*, and *Prevotella* were elevated significantly, while some probiotics, such as *Butyricicoccus*, *Clostridium*, *Lachnospiraceae*, *Lactobacillus*, and *Bifidobacterium* were decreased (Tao et al., 2017). The break-down of the intestinal flora caused by TNBS inevitably has an important impact on the development of IBD. Based on the above findings, in the present study, we tried to investigate the molecular mechanism of CP on the improvement of the colitis.

Toll-like receptors are the recognition receptors of the innate immune system, which locate on the surface of various immune cells of the body and play an important role in the defense of the pathogen infection and regulation of immune responses (Venkatesh et al., 2014). LPS, a typical bacterial endotoxin, is the major component of the cell wall of Gram-negative bacteria and is responsible for the activation of the innate immune system caused by bacterial infection (Chaluvadi et al., 2009; Sun et al., 2016). TLR4 usually is low expression in normal state of the body, while in IBD condition, the intestinal flora lose tolerance and specific recognition of LPS, then produce excessive signal transduction, and finally activate TLR4 and induce pro-inflammatory cytokines (Zawawi et al., 2010; Robertsthomson et al., 2011).

As a key transcription factor, NF-κB plays an important role in the activation of genes involved in immune and acute phase responses (Schottelius and Baldwin, 1999) and is an attractive candidate for its roles in IBD pathogenesis (Schottelius and Baldwin, 1999) which can increase the expression of many cytokines, enzymes, and adhesion molecules involved in chronic inflammatory diseases, including IL-1β, TNF-a, IL-2, IL-6, IFN-γ, and IL-12 which possess NF-κB binding sites and are transcriptionally regulated by NF-κB (Barnes and Karin, 1997; Schottelius and Baldwin, 1999). NF-κB in normal intestinal epithelial cells generally shows low expression in a certain range, which can be over activated in the inflammatory environment.

In this study, we demonstrated that TNBS up-regulated the protein expression and mRNA levels of TLR4 and NF-κB. Over expression of these signaling proteins promoted the secretion of cytokines, leading to the proliferation and metastasis of tumor cells (Huang et al., 2014; Wang et al., 2017). Compared with the normal group, mRNA levels of TLR4 and NF-κB in colitis rats were significantly increased, which were markedly down-regulated by different dosages of CP. An attenuated trend of the expression of p65, pp65, and TLR4 in TNBS-induced colitis rats was also observed in the groups treated with CP. Meanwhile, CP could notably down-regulate the levels of pro-inflammatory factors such as TNF-α, IFN-γ, IL-6, and IL-1β. These results suggested that CP exerted its effects on amelioration of TNBS-induced colitis via decreasing the expression of TLR4 and blocking the activation of NF-κB signaling pathways.

Lipopolysaccharide can activate multiple intracellular signal pathways, promote the expression of a large number of cytokines and adhesion molecules, and cause inflammation cascade reaction (Herzum and Renz, 2008; Zhao et al., 2015). IL-6 is a pleiotropic cytokine considered to be a major player in inflammation, regulation of T cell responses, and apoptosis (Kishimoto, 2005). IL-6-mediated signaling pathways are reported to play a critical role in the development of IBD (Atreya and Neurath, 2005). Combines to the receptor, IL-6 increased the phosphorylation of JAK2, an upstream activator of STAT3, and IL-6-induced STAT3 phosphorylation was obtained which regulated the downstream target gene transcription and participated in the regulation of inflammation (Lovato et al., 2003). Studies have shown that activated STAT3 was found in human and animal colitis models, and over expression of STAT3 was always accompanied by more serious of the disease state. IL-6-induced JAK2/STAT3 signaling pathways play an important role in the pathogenesis of IBD. Consistent with previous reports, we observed that TNBS-induced colitis invariably promoted inflammation in the colon with elevated levels of IL-1β, IL-6, TNF-α and an increased expression of JAK2, STAT3, and p-STAT3. In colitis rats, the observed up-regulation of pro-inflammatory cytokines including IL-6 was ameliorated by CP, meanwhile, phosphorylation of Stat3 and JAK2 was blocked. The results provided further evidences of the effects of CP on suppression of colitis and indicated the key role of IL-6/JAK2/STAT3 pathway as relevant targets of CP (Huo et al., 2016).

TLR4/NF-κB and IL-6/JAK2/STAT3 signal pathways were investigated to evaluate the alleviation of CP on the TNBS-induced colitis. It was documented that inhibition of STAT3 activation by a JAK2 inhibitor could reduce the nuclear pool of STAT3 resulting in a suppressed NF-κB activation (Lovato et al., 2012; Seavey et al., 2012). JAK2/STAT3 and NF-κB pathways regulated the expression of a wide spectrum of cytokines and chemokines in the tumor micro-environment, which controlled the activation of immune cells as well as growth and survival of premalignant and malignant epithelial cells, including IL-6, IL-1β, IL-11, IL-23, IFN-γ, TNF-α, and so on (Grivennikov et al., 2006). IL-6 has been shown to play a critical role in the development and progression of colitis and colitis-associated cancer. In our study, its levels in colon tissues were dramatically reduced by CP. Combinatorial effects of STAT3 and NF-κB inactivation could explain such a robust inhibition of IL-6 expression (Bollrath and Greten, 2009).

The results of plasma and urine metabolomics study of colitis rats indicated that endogenous metabolites of glycerophosphocholine and phosphorylcholine levels were decreased, which result in the metabolic disorder of phospholipid and choline metabolism in inflammation. The above two endogenous biomarkers are important metabolites of and the major cellular components of the biofilm, and their deletions lead to abnormalities in cell membrane biosynthesis. It is reported that the reduction of nucleotides and bases, such as nicotinic acid mononucleotides, dihydrouracil, could lead to the death of the colonic epithelial cells (Dong, 2013). The level of cholesterol in the plasma of the model group was increased significantly, which indicated the occurrence of abnormal lipid metabolism associated with colitis. Abnormal dyslipidemia is a typical clinical symptom in patients with the colitis which is closely related to the development of inflammation (Martin et al., 2009). The induction of TNBS/ethanol accelerated the expression of lipoprotein lipase and down-regulated the expression of CYP450 and CoA oxidase. Recent studies indicated that CYP450 enzymes played a significant physiological role in the body. They could control the intracellular cholesterol concentration and affect the transcription of low-density lipoprotein receptors and enzymes involved in cholesterol synthesis (Warren et al., 2001; Karlsson et al., 2008). The increase of cholesterol levels in plasma was accompanied by the TNBS-induced colitis due to the biosynthesis of fatty acids was consistent with our previous studies that the content of acetic acid in colon mucosa of colitis rats was decreased. Because acetic acid is easily converted into acetyl coenzyme A by cell uptake, it is the intermediate product of the synthesis of cholesterol and fatty acids (Yoshimoto et al., 2001). The down-regulation of nicotinamide and urocanic acid in the urine of colitis rats indicated that TNBS-induced ulcerative colitis rats reduced the antioxidant function and increased oxidative stress of the host, which was important reasons for the development and continuation of ulcerative colitis (Cho, 2008). Uric acid is produced by the deamination of L- histidine by histidinelyase. In the liver, the urocanic acid is converted to imidazol-4-oxo-5-propionic acid by the urocanic acid hydratase and eventually to glutamic acid, and subsequently, glutamic acid undergoes the decarboxylation by glutamic acid decarboxylase to form γ-aminobutyric acid, which is an important neurotransmitter and also precursor components of glutathione (Grisham, 1993). Compared with the normal group, the levels of niacinamide in TNBS-induced colitis was decreased. Studies have shown that nicotinamide enhanced the antimicrobial activity of neutrophil, improved tissue damage and reduced the activity of MPO of DSS-induced colitis mice significantly (Disher et al., 2014; Dominik et al., 2014). Consistent with previous studies, the levels of linoleic acid and arachidonic acid were decreased and the leukotriene A4 was increased in colitis rats. Arachidonic acid is an unsaturated fatty acid which is transformed from linoleic acid and linolenic acid, and could be converted into prostaglandins, leukotrienes and other inflammatory substances. IBD is bound to be closely related to the metabolic disorder of bile acids, especially to reduce the level of secondary bile acids, while hydrophilic secondary bile acids have cytoprotective effects. In this study, the down-regulation of taurodeoxycholic acid, chenodeoxycholic acid and bile acid in plasma of colitis rats was verified this point (Blackwood et al., 2011; Disher et al., 2014).

The metabolic pathways were established by importing the potential metabolites into the web-based database MetPA (**Figure 7**). Furthermore, the relative quantities of eight potential biomarkers in plasma and four in urine were significantly affected by CP. Especially, the middle (MP, 100 mg/kg) and low (LP, 50 mg/kg) dosage were restored to a control-like level. The results indicated that CP could notably alleviate the TNBS-induced colitis.

## Conclusion

The present study suggested that CP exerted a distinct inhibition effect on TNBS-induced colitis. CP could significantly reduce the levels of inflammatory cytokines, decrease mRNA levels of TLR4, NF-κB, IL-6, STAT3, and JAK2, and down regulate the expression of pp65, TLR4, p-STAT3, and p-JAK2. The anti-inflammation effect of CP in IBD might be associated with TLR4/NF-κB and IL-6/JAK2/STAT3 signaling pathway. Meanwhile, CP altered the biomarkers and the corresponding metabolic pathways in plasma and urine to normal transformation, which indicated its alleviation on TNBS-induced colitis.

## Ethics Statement

The animal experiments were performed according to the Regulations of Experimental Animal Administration (State Committee of Science and Technology of the People’s Republic of China). The guidelines of the Committee on the Care and Use of Laboratory Animals in China and the related ethics regulations of Nanjing University of Chinese Medicine. Laboratory animal license number is SYXK (Su)-2012-0042.

## Author Contributions

J-HT and J-AD conceived and designed the study. J-HT and D-DW performed the experiments. J-HT, SJ, and J-MG acquired the data. J-HT, J-AD, and J-MG analyzed the data. J-HT, J-AD, SJ, and WZ drafted and critically revised the article. J-HT, J-AD, WZ, SJ, J-MG, and D-DW approved the final manuscript.

## Conflict of Interest Statement

The authors declare that the research was conducted in the absence of any commercial or financial relationships that could be construed as a potential conflict of interest.

## Footnotes

1 http://metlin.scripps.edu

2 http://www.hmdb.ca

3 http://www.metaboanalyst.ca/

4 http://www.genome.jp/kegg/

5 http://www.genome.jp/kegg/

6 http://www.hmdb.ca

## Supplementary Material

FIGURE S1 Effects of CP on colitis rats. **(A)** Change in body weight during the disease. **(B)** DAI based on weight loss, hematochezia, and diarrhea. **(C)** Statistics of colon length in each group. **(D)** Histopathological changes in the colons.

FIGURE S2 Ultra-performance liquid chromatography/quadrupole time of flight mass spectrometry (UPLC-TOF/MS) TIC chromatograms of plasma and urine sample in normal and colitis rats. **(A)** Plasma; **(B)** Urine.

TABLE S1 Primer sequences of the target gene.

## Abbreviations

CP: Chrysanthemum polysaccharides
HP: TNBS/ethanol + high dosage CP
IBD: inflammatory bowel disease
JAK2: janus kinase 2
LP: TNBS/ethanol + low dosage CP
LPS: lipopolysaccharide
M: TNBS/ethanol group
MDA: malondialdehyde
MP: TNBS/ethanol + middle dosage CP
MPO: myeloperoxidase
MyD88: myeloid differentiation factor 88
N: Normal group
NF-κB: nuclear factor kappa-B
PCA: principal component analysis
PLS-DA: partial least squares discriminant analysis
PP: TNBS/ethanol + Sulfasalazine
PRRs: pattern recognition receptors
SOD: superoxide dismutase
STAT3: signal transduction and transcriptional activators
TBA: thiobarbituric acid
TLRs: toll-like receptors
TNBS, 2, 4: 6-trinitrobenzene sulfonic acid
UPLC-Q-TOF/MS: ultra-performance liquid chromatography/quadrupole time of flight mass spectrometry
VIP: variable importance.
