# Supplementary figures and images for: Polysaccharides From Chrysanthemum morifolium Ramat Ameliorate Colitis Rats via Regulation of the Metabolic Profiling and NF-κ B/TLR4 and IL-6/JAK2/STAT3 Signaling Pathways
Source: Front Pharmacol. 2018 Jul 10;9:746. doi: 10.3389/fphar.2018.00746 (PMC6049019; doi:10.3389/fphar.2018.00746)

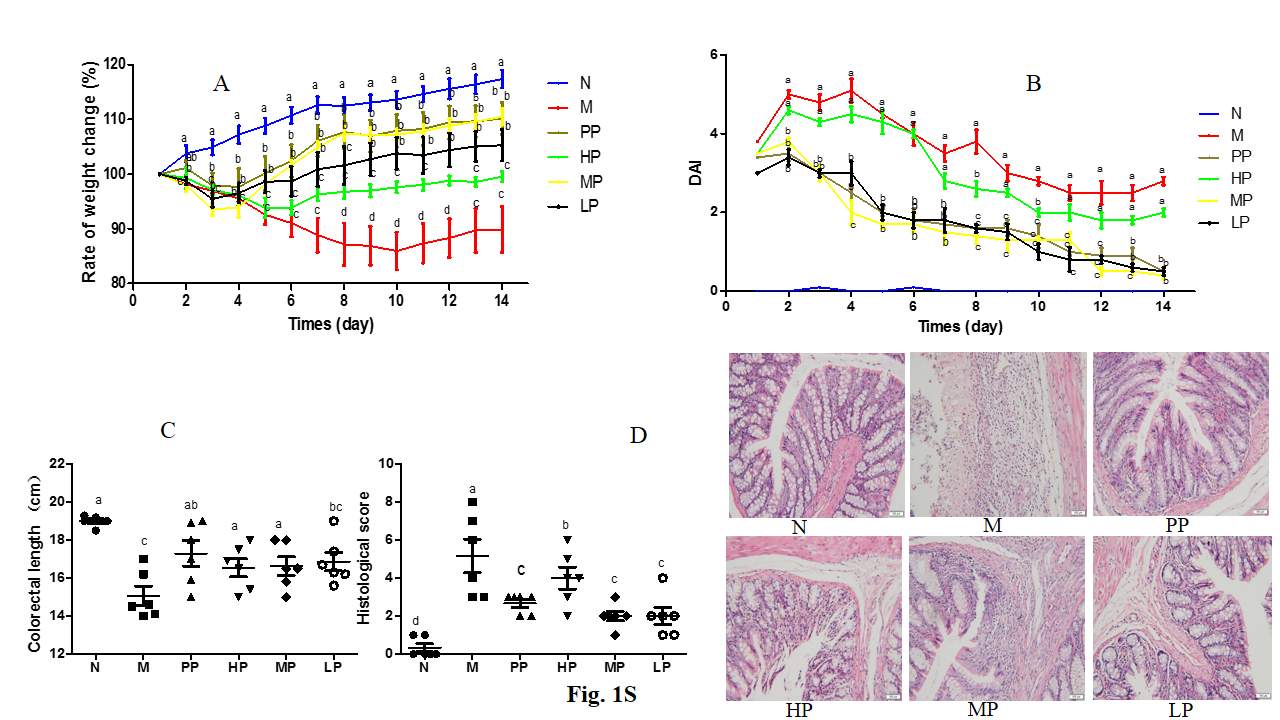

Supplement: FIGURE S1 — Effects of CP on colitis rats. (A) Change in body weight during the disease. (B) DAI based on weight loss, hematochezia, and diarrhea. (C) Statistics of colon length in each group. (D) Histopathological changes in the colons. [file Image_1.TIF]

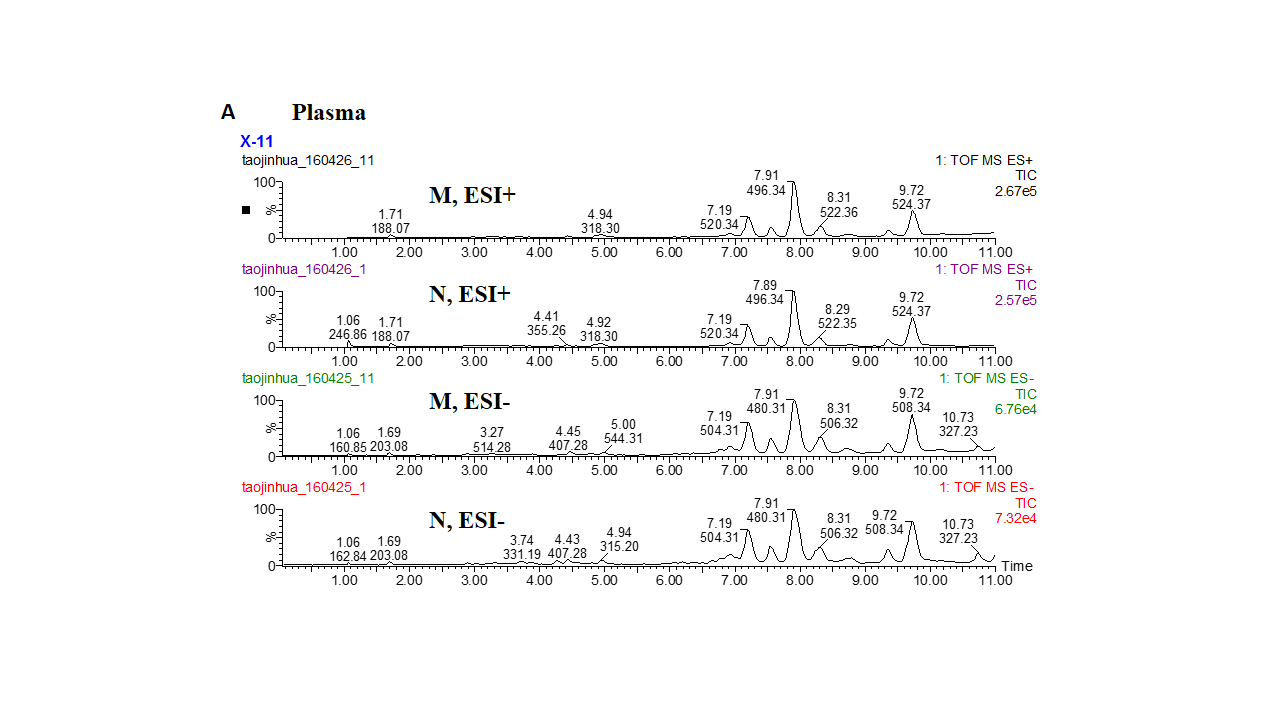

Supplement: FIGURE S2 — Ultra-performance liquid chromatography/quadrupole time of flight mass spectrometry (UPLC-TOF/MS) TIC chromatograms of plasma and urine sample in normal and colitis rats. (A) Plasma; (B) Urine. [file Image_2.TIF]

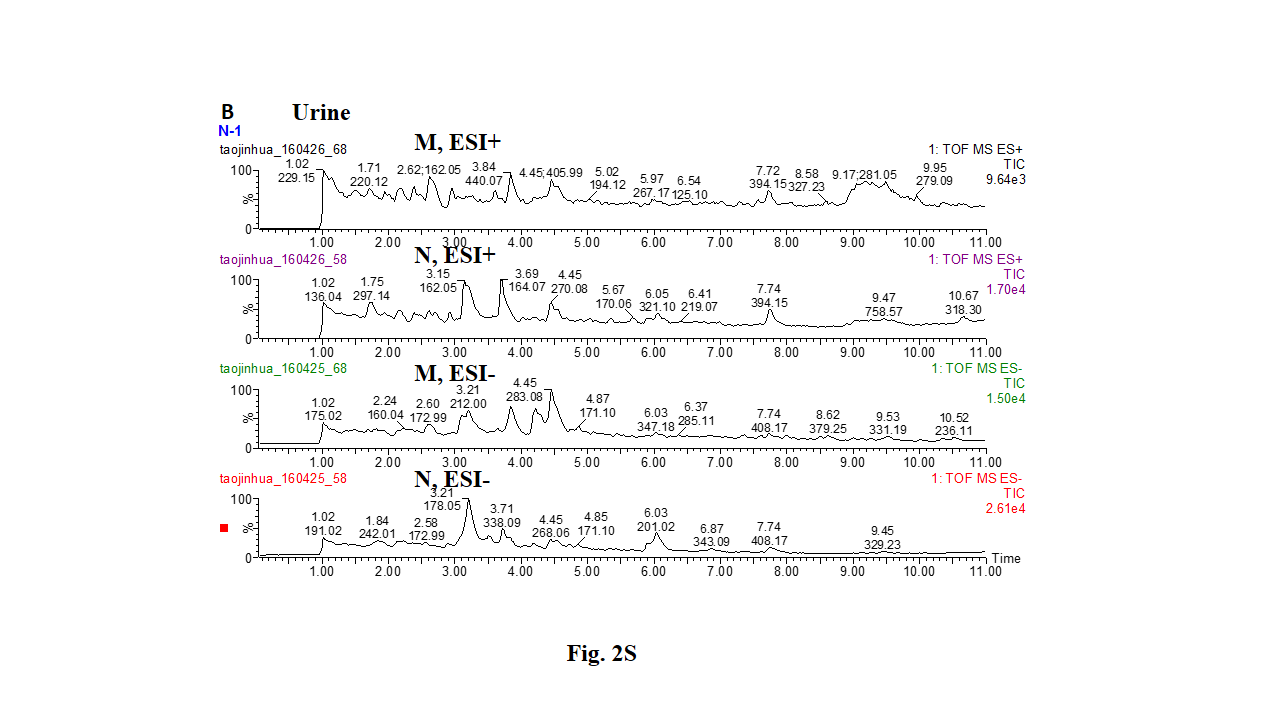

Supplement: Supplementary file 3 [file Image_3.TIF]
