# Supplementary material for: Polysaccharides From Chrysanthemum morifolium Ramat Ameliorate Colitis Rats via Regulation of the Metabolic Profiling and NF-κ B/TLR4 and IL-6/JAK2/STAT3 Signaling Pathways
Source: Front Pharmacol. 2018 Jul 10;9:746. doi: 10.3389/fphar.2018.00746 (PMC6049019; doi:10.3389/fphar.2018.00746)
Supplement: TABLE S1 — Primer sequences of the target gene. [file Table_1.PDF]

Table 1S Primer sequences of the target gene

**Gene**	Primer sequences		Amplification length
IL-6	Forward primer	GAAATGTGGTCGGCAAGTCC	110
	Reverse primer	CTGGGATGCAGGGTGAGTTC	
NF-kB	Forward primer	CAAGATCTGCCGAGTAAACC	201
	Reverse primer	TCGGAACACAATGGCCACTT	
TNF-a	Forward primer	CGGTGCCTATGTCTCAGCCT	109
	Reverse primer	TCCAGCTGCTCCTCCACTTG	
IL-1β	Forward primer	AGCCAACAAGTGGTATTCTCC	168
	Reverse primer	TGAAGACAAACCGCTTTTCCA	
TLR4	Forward primer	GACCTCAGCTTCAATGGTGTC	199
	Reverse primer	TCAAGCCAAGAAATATGCCATC	
β-actin	Forward primer	GAC CCA GAT CAT GTT TGA GAC	204
	Reverse primer	GTA GCC ACG CTC GGT CAG	
GAPDH	Forward primer	ACA TCA AGA AGG TGG TGA AGC	108
	Reverse primer	TTG TCA TAC CAG GAA ATG AGC TT	
