# Supplementary material for: Polysaccharides From Chrysanthemum morifolium Ramat Ameliorate Colitis Rats via Regulation of the Metabolic Profiling and NF-κ B/TLR4 and IL-6/JAK2/STAT3 Signaling Pathways
Source: Front Pharmacol. 2018 Jul 10;9:746. doi: 10.3389/fphar.2018.00746 (PMC6049019; doi:10.3389/fphar.2018.00746)
Supplement: Supplementary file 5 [file Presentation_1.PDF]

Induction of colitis rats model and treatment protocol

Acute colitis rats were induced by rectal administration of TNBS mixed with a certain percentage of ethanol through a special catheter (Scheiffele *et al.*, 2002). Briefly, rats were anaesthetized with 10% chloral hydrate, and subsequently administered with 3 mL/kg of TNBS-ethanol solution (50 mg/mL) into the colon at 8 -10 cm depth from the rectum using a soft polyethylene catheter. The rats were fastened in the trendelenburg position for one minute to avoid loss of TNBS solution via the rectum. While normal rats were rectally administered with normal saline at equivalent instead of TNBS (Yang *et al.*, 2014). 24 hours (day 1) after induction of colitis, all the rats were randomly assigned to six groups, five rats were chosen in each group: Normal group (N) , receiving normal saline at equivalent and received intragastric administration (ig) saline during treatment; TNBS model group (M), receiving ethanol vehicle with TNBS (TNBS + saline); Sulfasalazine group (PP), receiving SASP 0.5g/kg (TNBS + SASP); Chrysanthemum polysaccharides (CP) high, middle and low dose treatment group (HP, MP, LP), receiving CP 200 mg/kg (TNBS + HP)， 100 mg/kg (TNBS + MP) and 50 mg/kg (TNBS + LP), respectively. All above treatments were from day 2 to day 15. The rats were detected daily for colitis by clinical symptoms including bodyweight, gross rectal bleeding and stool consistency, which were assessed by DAI according to the method described by Cooper (Cooper *et al.*, 1993).

Amelioration of Chrysanthemum polysaccharides on TNBS-induced colitis

In this study, intra-colonic instillation of TNBS-induced colitis rat model was established and successfully applied to evaluate the amelioration of Chrysanthemum polysaccharides. From the fourth day of rectal administration of TNBS, rats showed increasingly severe symptoms such as serious diarrhea, obvious rectal bleeding and notable body weight loss. Compared to the normal group (Figure 1S-A), TNBS-induced colitis rats (M) remarkably lost weight throughout the trial period ($p < 0.01$), which was rescued by the Chrysanthemum polysaccharides treatment (HP 200 mg/kg, MP 100 mg/kg, LP 50 mg/kg). Disease activity index (DAI) was prominently higher in the model group than that in the normal group ($p < 0.01$). Compared with

the model group, treatments with low and middle doses of Chrysanthemum polysaccharides and SASP notably reduced DAI ($p < 0.01$) (Figure 1S-B). Shortened colon length is an important physiological index of colitis. TNBS treated rats showed substantial reduction in colon length compared with the normal group ($p < 0.001$). Chrysanthemum polysaccharides at 50 mg/kg alleviated the situation of colon shortening (Figure 1S-C) ($p < 0.05$). The histopathological characteristics of colon tissues from each group were evaluated by H&E staining. The results indicated that severe pathological changes such as mucosal lesion, necrosis and infiltration of inflammatory cells including monocytes and neutrophils occurred in the colonic tissues of model rats, which were alleviated by Chrysanthemum polysaccharides treatment, especially the MP (100 mg/kg) and LP (50 mg/kg) ($p < 0.01$) (Figure 1S-D).

References

Cooper HS, Murthy SN, Shah RS (1993). Clinicopathologic Study of Dextran Sulfate Sodium Experimental Murine Colitis. Laboratory Investigation; a Journal of Technical Methods and Pathology, 69: 49-238.

Scheiffele F, Fuss IJ (2002). Induction of TNBS Colitis in Mice. Current Protocols in Immunology, Chapter 15.19.1-15.19.14.

Yang M, Lin HB, Gong S (2014). Effect of Astragalus Polysaccharides on Expression of TNF-α, IL-1β and NFATc4 in a Rat Model of Experimental Colitis. Cytokine, 70: 81-86.
